# The bacterial protein toxin, cytotoxic necrotizing factor 1 (CNF1) provides long-term survival in a murine glioma model

**DOI:** 10.1186/1471-2407-14-449

**Published:** 2014-06-18

**Authors:** Eleonora Vannini, Anna Panighini, Chiara Cerri, Alessia Fabbri, Simonetta Lisi, Enrico Pracucci, Nicola Benedetto, Riccardo Vannozzi, Carla Fiorentini, Matteo Caleo, Mario Costa

**Affiliations:** 1 CNR Neuroscience Institute, Via Moruzzi 1, 56124 Pisa, Italy; 2 Scuola Normale Superiore, Piazza Dei Cavalieri 7, 56100 Pisa, Italy; 3 Istituto Superiore di Sanità, Viale Regina Elena 299, 00161 Rome, Italy; 4 Neurochirurgia, Azienda Ospedaliero-Universitaria Pisana, Via Paradisa 2, 56100 Pisa, Italy

**Keywords:** Glioma, Mouse, Cerebral cortex, CNF1, Temozolomide

## Abstract

**Background:**

Glioblastomas are largely unresponsive to all available treatments and there is therefore an urgent need for novel therapeutics. Here we have probed the antineoplastic effects of a bacterial protein toxin, the cytotoxic necrotizing factor 1 (CNF1), in the syngenic GL261 glioma cell model. CNF1 produces a long-lasting activation of Rho GTPases, with consequent blockade of cytodieresis in proliferating cells and promotion of neuron health and plasticity.

**Methods:**

We have tested the antiproliferative effects of CNF1 on GL261 cells and human glioma cells obtained from surgical specimens. For the *in vivo* experiments, we injected GL261 cells into the adult mouse visual cortex, and five days later we administered either a single intracerebral dose of CNF1 or vehicle. To compare CNF1 with a canonical antitumoral drug, we infused temozolomide (TMZ) via minipumps for 1 week in an additional animal group.

**Results:**

In culture, CNF1 was very effective in blocking proliferation of GL261 cells, leading them to multinucleation, senescence and death within 15 days. CNF1 had a similar cytotoxic effect in primary human glioma cells. CNF1 also inhibited motility of GL261 cells in a scratch-wound migration assay. Low dose (2 nM) CNF1 and continuous TMZ infusion significantly prolonged animal survival (median survival 35 days vs. 28 days in vehicle controls). Remarkably, increasing CNF1 concentration to 80 nM resulted in a dramatic enhancement of survival with no obvious toxicity. Indeed, 57% of the CNF1-treated animals survived up to 60 days following GL261 glioma cell transplant.

**Conclusions:**

The activation of Rho GTPases by CNF1 represents a novel potential therapeutic strategy for the treatment of central nervous system tumors.

## Background

Gliomas are primary central nervous system tumors that arise from astrocytes, oligodendrocytes or their precursors. Following the World Health Organization (WHO) classification, gliomas can be classified in 4 groups according to their histological characteristics and the most malignant form is glioblastoma multiforme (GBM). GBM is uniformly fatal and largely unresponsive to all available treatments. Despite intensive therapy including surgery, radiotherapy and chemotherapy, the average of survival of patients with glioblastoma usually is 15 months from the time of first diagnosis [1,2]. Conventional surgical excision, generally limited to the main tumor mass, does not remove the microscopic foci of neoplastic cells that invade the surrounding normal brain substance beyond the main tumor mass, and that are responsible for the inevitable tumor recurrence. Radiotherapy and chemotherapy, often associated to surgery, cannot ablate completely these tumors, since this would require unacceptably high radiation/chemotherapic doses that result in severe brain-neuron damage. There is therefore a clear need to accelerate progress in the development of new strategies for treatment of glioma.

Several therapeutic approaches for glioma are currently being investigated in animal models and patients. They include delivery of cytotoxic genes and proteins to glioma cells, suppression of angiogenesis, and immune stimulation [3]. Concerning chemotherapy, alkylating agents such as temozolomide (TMZ) are widely used in the treatment of brain tumours [4]. As a cytotoxic alkylating agent, TMZ is converted at physiologic pH to the short-lived active compound, monomethyl triazeno imidazole carboxamide (MTIC). The cytotoxicity of MTIC is primarily due to methylation of DNA at the O6 and N7 positions of guanine, resulting in inhibition of DNA replication. Chemotherapics have substantial side effects and limited efficacy, and this further underlies the need of innovative approaches for glioma treatment.

In this paper we describe a potential novel therapy for glioma, based on intracerebral administration of cytotoxic necrotizing factor 1 (CNF1), a bacterial protein toxin produced by specific strains of *Escherichia coli*. CNF1 is a single-chain protein, consisting of a N-terminal domain involved in cell binding, a middle region mediating membrane translocation, and a C-terminal catalytic domain. The C-terminal part of CNF1 is released into the cytosol where it catalyzes the deamidation of a single glutamine residue of the Rho GTPases (RhoA, Rac1 and Cdc42). Rho GTPases are molecular switches that cycle between a GDP-bound inactive and a GTP-bound active state to control a multitude of cellular events, like actin cytoskeleton organization as well as gene transcription, cell proliferation, and survival [5]. Rho GTPases deamidated by CNF1 are not able to hydrolyse GTP and remain in a persistent activated state [6,7] which is followed by partial deactivation of these regulatory proteins via degradation by the ubiquitin–proteasome pathway [8]. The persistent activation of Rho GTPases by CNF1 causes a remarkable reorganization of the actin cytoskeleton with dramatic functional consequences. In particular, cultured proliferating cells exposed to CNF1 acquire a multinucleated phenotype (cytotoxic effect), due to stabilization of the actin network and prevention of cytodieresis despite ongoing nuclear division [9]. On the other hand, our recent studies have demonstrated “plasticizing” effects of CNF1 in neurons. Specifically, intracerebral administration of CNF1 improves neuronal function, learning and memory [10,11], and these effects are associated with a enhancement of brain plasticity, exemplified by the increase in spine density in cortical neurons [10].

In view of these striking, differential effects of CNF1 on proliferating cells and neurons, we have probed for the first time the potential antitumoral effects of this toxin in cell culture and in an animal model. Among the available glioma models, we adopted the well accepted GL261 syngeneic mouse model of high grade glioma [12,13] based on intracerebral injection of GL261 cells in C57/Bl6 mice. We demonstrate the cytostatic and cytotoxic effects of CNF1 on GL261 glioma cell line, and the survival-promoting effect on tumor-bearing animals. We have also provided proof-of-principle for cytotoxic effects of CNF1 in human tumor cells.

## Methods

### GL261 glioma cell cultures

The murine glioma GL261 cell line was a kind gift from Dr. C. Sala (CNR Neuroscience Institute, Milan). GL261 cells were grown in complete Dulbecco’s modified Eagle’s medium (DMEM) containing 10% Newborn calf serum, 4.5 g/L glucose, 2 mM glutamine, 100 UI/ml penicillin and 100 mg/ml streptomycin at 37°C in 5% CO_2_ with media changes three times per week.

### CNF1

*E. coli* CNF1 was obtained from the 392 ISS strain (kindly provided by V. Falbo, Istituto Superiore di Sanità, Rome, Italy) and purified as described previously [14]. The levels of lipopolysaccharide (LPS) in the CNF1 preparation were assessed by the Limulus Amebocyte Lysate (LAL) kinetic chromogenic assay (performed by LONZA Verviers Sprl). The LPS concentration determined by the assay (0.07 ng/ml) was much lower than that required to achieve biological effects (e.g. 1 ng/ml in macrophages, one of the most sensitive cells to LPS).

The activity of every batch of CNF1 was tested on GL261, treating the cells for 24 hours. Three parameters were considered: i) cells morphology (enlargement and flattening of cells), ii) increased size of nucleoli and iii) the ratio between mono and multinucleated cells. The activity of each CNF1 preparation was considered satisfactory if at least one of the three parameters indicated above were present in more than 60% of treated cells.

### Clonogenic assay

GL261 cells were harvested by trypsinization, counted and seeded onto twenty four-well plates at a density of 300 cells/plate. To assess the effect of CNF1 on cell proliferation, GL261cells were incubated for 9 days with different concentrations of CNF1 (from 8 × 10^−11^ to 3.2 × 10^−9^ M). Nine days after treatment, cells were stained with crystal violet, the number of colonies containing at least 50 cells was counted [15] and the effective half inhibitory dose (IC50) of the toxin was calculated on the basis of linear regression equation.

### Wound healing-migration assay

The wound healing migration assay was performed according to Liang [16] with minor modifications. Briefly, GL261 cells were seeded in 6-well cell plates and cultured to a confluent monolayer. A sterile pipette tip (200 μl) was used to make a straight scratch on the monolayer of cells and the wound was allowed to heal for 8, 24 and 48 hours. To evaluate CNF1 effects, GL261 cells were incubated with CNF1 for 24 hours before making the scratch, and wound healing was assessed 8, 24 and 48 hours after the injury. The cells were then fixed with methanol and stained with crystal violet. The extent of cell migration was photographed and the wound size measured using image analysis software (ImageJ).

### Apoptosis-necrosis assays

Apoptotic and/or necrotic cells were determined using Annexin V- Propidium Iodide (PI) Staining Kit. The assay was performed following the manufacturer instructions (Annexin V kit, BD Pharmingen). Briefly, 300 GL261 cells were seeded on twenty four-well plates and incubated in CNF1 (18 nM) for 10 days. Annexin V and Propidium Iodide were diluted 1:200 in KREB medium (NaCl 120 mM, NaKCO_3_ 25 mM, KCl 4.7 mM, KH_2_PO_4_ 1.18 mM, MgSO_4_ 1.18 mM, CaCl_2_ 2.5 mM, EDTA 0.026 mM, glucose 5.5 mM). Cells were also stained with Hoechst dye (bisbenzimide, Sigma; 1:500 in PBS) and counted with fluorescence microscopy. We classified cells as positive for annexin V only (Ann V + PI-), positive for PI only (Ann V- PI+), positive for both markers (Ann V + PI+) or unlabeled.

### Senescence-Associated Beta-Galactosidase (SA beta-gal) Assay

To determine cellular senescence, GL261 cells were plated in triplicate at low density (50% confluence) in 24-well plates. The cells were treated with CNF1 (1 nM) and incubated for 24, 48 or 72 hours before beta-galactosidase measure (senescence detection kit, Abcam catalog ab65351). The percentage of positively stained cells was determined after counting three random fields of cells. Representative microscopic fields were photographed under a 20× objective.

### Human glioblastoma cell cultures

Human biopsies of glioblastoma multiforme (GBM) were collected from 2 subjects who underwent brain surgery for tumor removal. The study was approved by the Human Ethics Committee of the University of Pisa and Pisa Hospital. Written, informed consent was obtained from the patients according to institutional guidelines. Patient 1 had a left parietal lesion resembling a high grade glioma on a contrast enhanced MRI scan, while patient 2 had a similar lesion located in the left fronto-parietal region. A surgical gross total resection was performed and the histological examination confirmed the suspected tumor type (WHO grade IV) in both cases. After collection, primary tumor cells were isolated according to [17]. Briefly, tissue explants were incubated with trypsin in DMEM for 10 minutes at 37°C and then added of 3 volumes of DMEM with 10% FBS. Tissue was completely disgregated by gentle pipetting. Cells suspension was then plated on tissue culture dishes and culture medium (DMEM, 10%FBS, 100 IU/ml penicillin,100 mg/ml streptomycin) was replaced every 3–5 days. After a week cells were trypsinized and splitted. At passage five [18] cells were plated on cover slips and then treated with 18nM CNF1 for 9 days. At this time point, cells were fixed and stained to observe their morphology.

### Animals and tumor induction

To induce glioma formation, C57BL/6 mice (12–14 weeks old) received a stereotaxically guided injection of 40,000 GL261 cells (20,000 cells/μl PBS solution) into the visual cortex (2 mm lateral to the midline and in correspondence with lambda) using fine glass micropipettes (tip diameter 40 μm). All experimental procedures were in conformity to the European Communities Council Directive 86/609/EEC. The animal experiments described in this manuscript have been approved by the Italian Ministry of Health, Department of Animal Health (Office n. 6), with decree #258-2012/B, dated Oct 23, 2012.

### CNF1 injections and TMZ minipumps

Five days after GL261 cells injection (tumor induction), mice were divided into three groups. The first group received CNF1 injection, the second Tris–HCl buffer injection (control condition). Stereotaxic infusions of CNF1/Tris–HCl (1 μl of a 2 or 80 nM solution) were made into the primary visual cortex of adult mice under avertin anesthesia (intraperitoneal injection of 2,2,2-tribromoethanol solution; 0.1 ml/5 g body weight). Injections were performed in three sites: 1.5, 2 and 2.5 mm lateral to the midline in correspondence with lambda. CNF1/Tris–HCl injections was slowly delivered at a depth of 0.7–0.8 mm from the pial surface.

The third experimental group received Temozolomide (TMZ) micropumps implantation for a week. Minipump implantation was performed as described previously [19,20]. Micro-osmotic pumps (Alzet 1007D; Alza, USA; pumping rate 0.5 μl/hr) were filled with TMZ (20, 140 and 200 μM solution) and connected with polyethylene tubing to 30 G stainless steel cannulae [20]. A small hole was made in the skull (2 mm lateral, 1 mm anterior) and the cannula was lowered into the cortex. The minipump was positioned subcutaneously under the neck and the cannula was secured to the skull with acrylic cement. Animals survival was checked daily and monitored up to 60 days after tumor implantation. At this time, mice that were still alive were deeply anesthetized and perfused with 4% paraformaldehyde. Coronal brain sections through the occipital cortex (45 μm thick) were cut with a freezing microtome, stained with Hoechst dye (1:500, Sigma) and histopatological examinations were carried out.

### Statistical analysis

Statistical analysis was performed with SigmaPlot (version 11). Differences between three or more groups were evaluated with one way analysis of variance (ANOVA) followed by Holm-Sidak test.

Survival analysis was performed using Kaplan-Meier (LogRank) statistics.

## Results

### CNF1 blocks GL261 cell proliferation in culture

**Figure 1:**
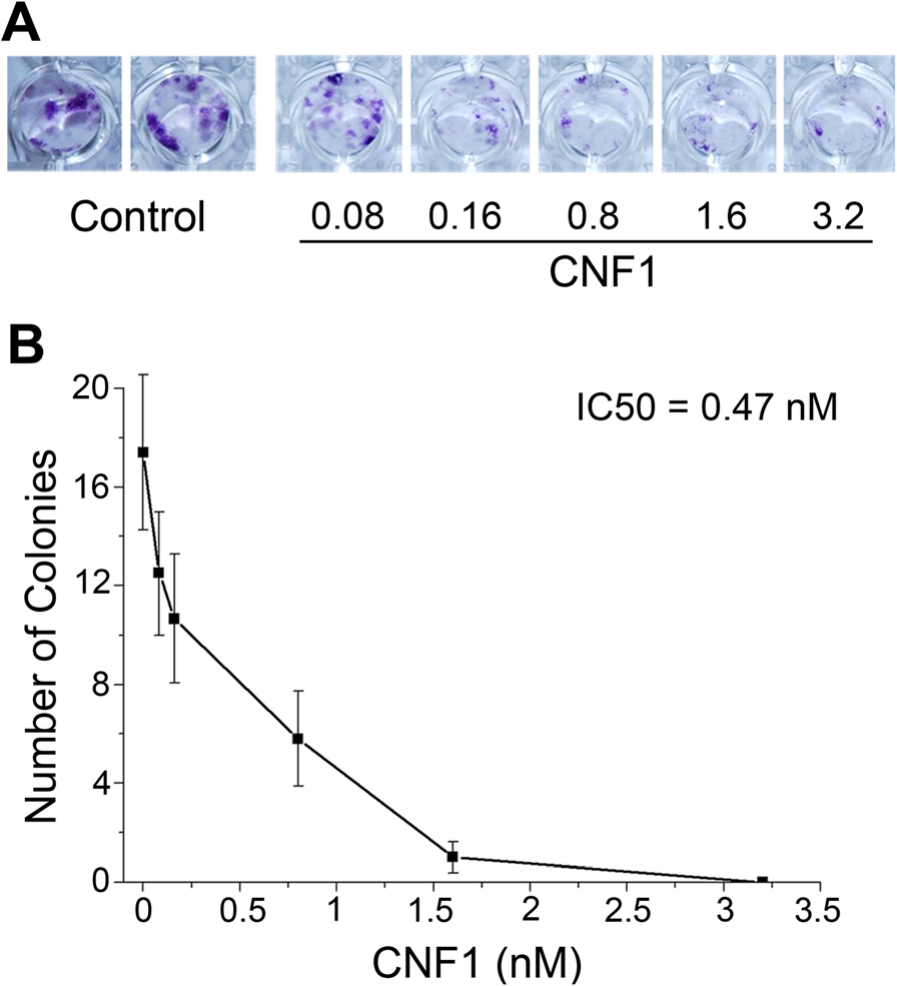
**Clonogenic assay and inhibitory effect of CNF1 on GL261 cells proliferation. (A)** Representative images of a colony assay of cells treated with vehicle (control) and increasing concentrations of CNF1. **(B)** Quantification of the number of colonies in the clonogenic assay for each CNF1 concentration. IC50 of the CNF1 toxin is 0.47 nM. All data represent means ± SEM (n = 3).

We first used a clonogenic assay to determine whether glioma cell proliferation is affected by CNF1. GL261 cells were plated at low density and exposed to different concentrations of CNF1. Colonies were counted after 9 days. We found that CNF1 inhibits colony formation in a dose-dependent manner (Figure 1). The half maximal inhibitory concentration (IC50) of CNF1 was found to be 0.47 nM (Figure 1B). In subsequent experiments, we always used CNF1 concentrations in the nanomolar range to ensure robust effects on cell proliferation.

### A short exposure time is sufficient for CNF1 to exert its effects

**Figure 2:**
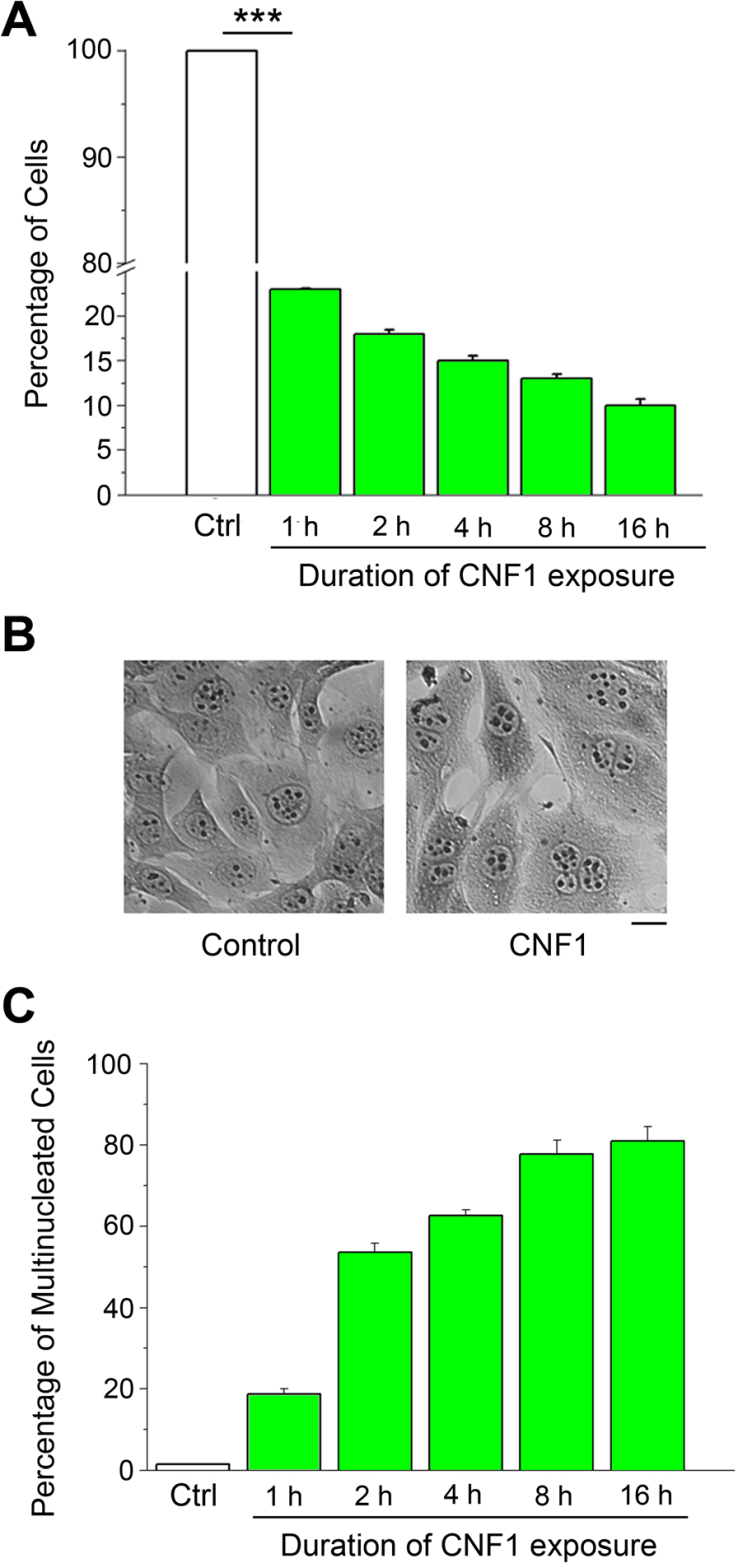
**A brief CNF1 exposure is sufficient to stop cell proliferation. (A)** Percentage of living GL261 cells after different time periods of CNF1 exposure. Data are normalized to the values obtained in vehicle-treated control (Ctrl) cells. **(B)** Crystal violet staining showing multinucleation of GL261 cells after 16 hours CNF1 exposure. Scale bar = 4 μm **(C)** Relative percentage of multinucleated cells in dishes treated with vehicle (Ctrl) and CNF1 for different periods. All data represent means ± SEM (n = 3).

In order to determine the minimal exposure time required by CNF1 to exert its effects, cells were treated with CNF1 (18 nM) for different times (ranging from 1 up to 16 hours). At the end of each incubation time, the toxin was replaced with fresh medium. Effects of the toxin were evaluated by counting cell numbers five days after treatment. The experiments demonstrated that 1 hour exposure to CNF1 was sufficient to stop proliferation, as shown by the dramatic (about 80%) reduction in cell numbers as compared to control (no treatment) conditions (one way ANOVA, p < 0.001; post hoc Holm-Sidak test, p < 0.001; Figure 2A). CNF1 effects became even more prominent by increasing the exposure time to 16 hours (p < 0.001; Figure 2A). We also evaluated the morphological appearance of the treated cells (Figure 2B). We found that in control conditions all cells were mononucleated, while in cells treated with CNF1 for 16 hours this phenotype decreased to 16%, and the percentage of multinucleated cells increased with longer CNF1 exposures (one way ANOVA, p < 0.001; post hoc Holm-Sidak test, p < 0.001; Figure 2C).

### CNF1 reduces the migration capability of GL261 cells

**Figure 3:**
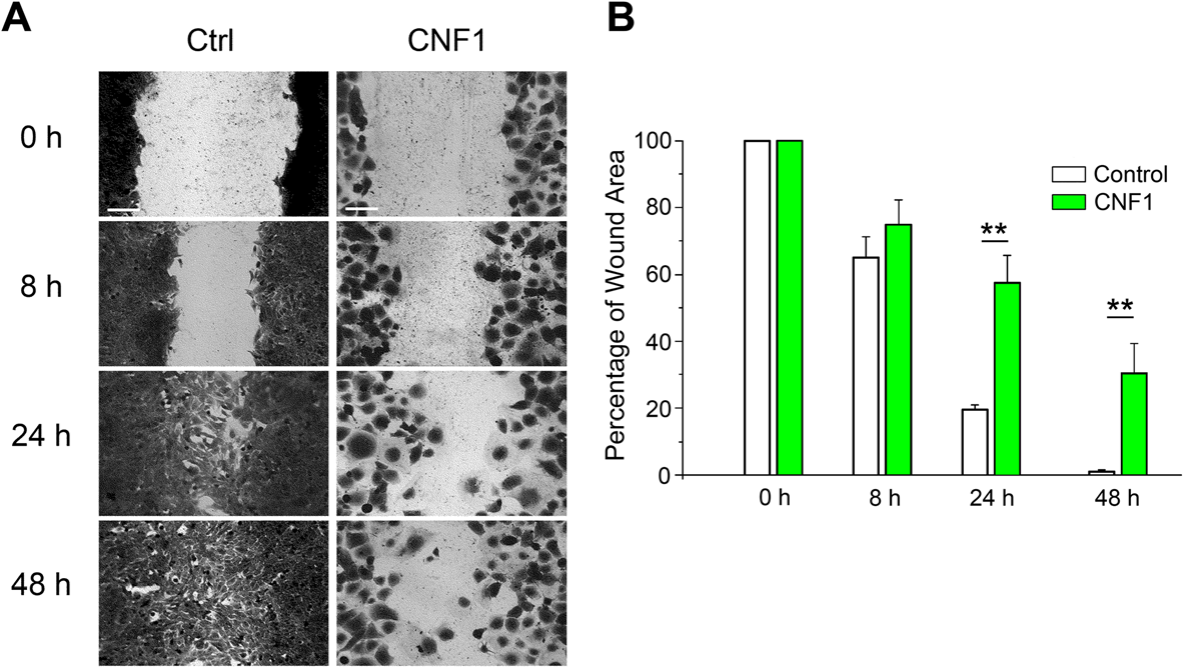
**Wound-healing migration assay of CNF1-treated Gl261 cells. (A)** Representative images of wound in untreated cells (left column) and CNF1-treated cells (right column) at different time points (0, 8, 24 and 48 hours). The data demonstrate the inability of CNF1-treated cells to invade the wound area. **(B)** Quantitative plots of the wound size at different time points in the two conditions (CNF1 and control). Data shown are representative of three independent experiments. All data represent means ± SEM. (two-way ANOVA, **p < 0.001). Scale bar = 100 μm.

A wound healing assay was performed in the GL261 cells incubated in culture medium with or without CNF1 (1 nM) to observe the effect of CNF1 on the migration. The area that the cells had migrated within 8, 24 and 48 hours (toward the initially scratched midline, from the border line) was measured. The experiments demonstrated that after 8 hours the number of cells migrating in the wound increased in normally growing cells, whereas fewer cells migrated in the wound area in CNF1-treated cells. The treatment effect was more evident after 24 hours, when in control cells the wound was healed to 80% whereas in CNF1-treated cells it was closed to about 30% and, at 48 hours, in control the wound was totally closed while in treated cells was half closed (Figure 3A and B).

### Fate of GL261 cells after prolonged CNF1 exposure

**Figure 4:**
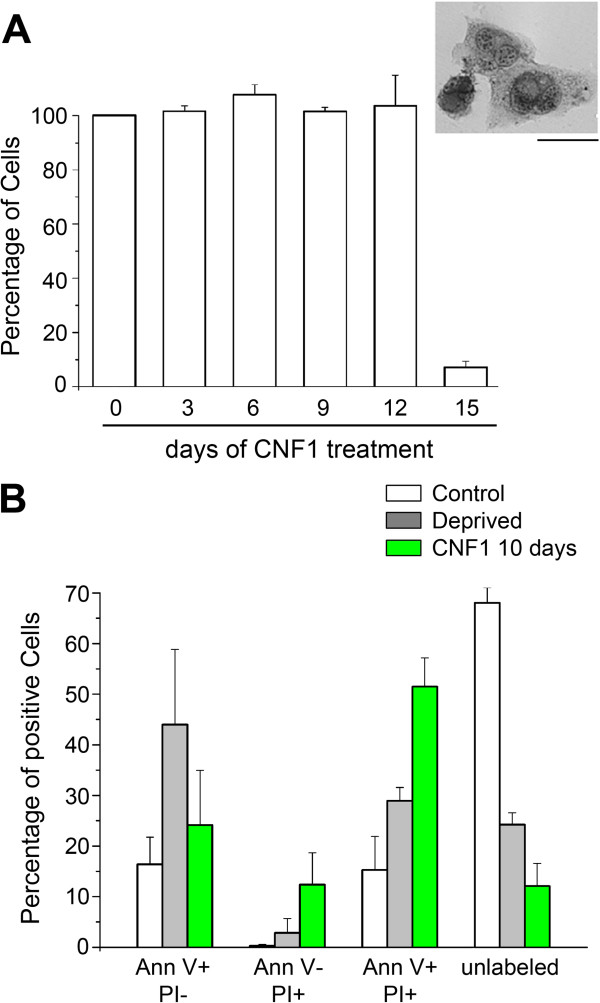
**Cytotoxic effect of CNF1 on GL261 cells. (A)** Percentage of living GL261 cells after continuous exposure to CNF1 (18 nM). Data represent means ± SEM (n = 3). Inset: Morphology of GL261 cells treated with CNF1 for 9 days. Scale bar = 5 μm. **(B)** Percentage of cells stained only by Annexin V (Ann V + PI-, left columns), stained only by Propidium Iodide (Ann V- PI+), positive for both markers (Ann V + PI+) and unlabeled (right columns). The analysis was performed in untreated GL261 cells (Control, open bars), in GL261 cells serum deprived for 2 days (grey bars), and in GL261 cells treated with CNF1 for 10 days (green bars). CNF1 induces a very significant upregulation in the frequency of Annexin V-Propidium Iodide double labelled cells (one way ANOVA followed by Holm-Sidak test, p < 0.05).

To determine the fate of polynucleated GL261, cells were treated with CNF1 (18 nM) at day one and then cultured without removal of the toxin for 15 days. We found a clear cytostatic effect of CNF1 until day 12 (Figure 4A). At this time point, GL261 cells treated with CNF1 still showed the characteristic altered morphology (enlargement and flattening of cells, increased size of nucleoli and multinucleation; see also Methods; Figure 4A, inset). Importantly, treatment for 15 days resulted in no survival of GL261 cells (Figure 4A), consistent with a cytotoxic action of CNF1.As a first step towards understanding the mechanisms of CNF1-induced GL261 cell death, we stained cells with markers of apoptosis and necrosis (annexin V and propidium iodide, respectively) 10 days after treatment. We chose this time point as it corresponds to a stage that just precedes cell disappearance, however all cells are still attached to dish and show the morphological features of senescence. As a positive control for apoptosis, we used GL261 cells deprived of serum for 2 days, which showed a robust increase in annexin V, but not PI staining (Figure 4B, left columns, AnnV + PI-; one way ANOVA followed by Holm-Sidak test, p < 0.05; Figure 4B, grey bars). GL261 cells treated with CNF1 for 10 days showed no clear evidence for either apoptotic (Figure 4B, left columns, “AnnV + PI-“) or necrotic cell death (Figure 4B, “AnnV- PI+”). However, more than 50% of CNF1-treated cells at 10 days resulted positive for both markers (Figure 4B, “AnnV + PI+”; one way ANOVA followed by Holm-Sidak test, p < 0.05) indicating profound cellular distress and late stages of cell demise.

### CNF1 induces early activation of senescence pathway

**Figure 5:**
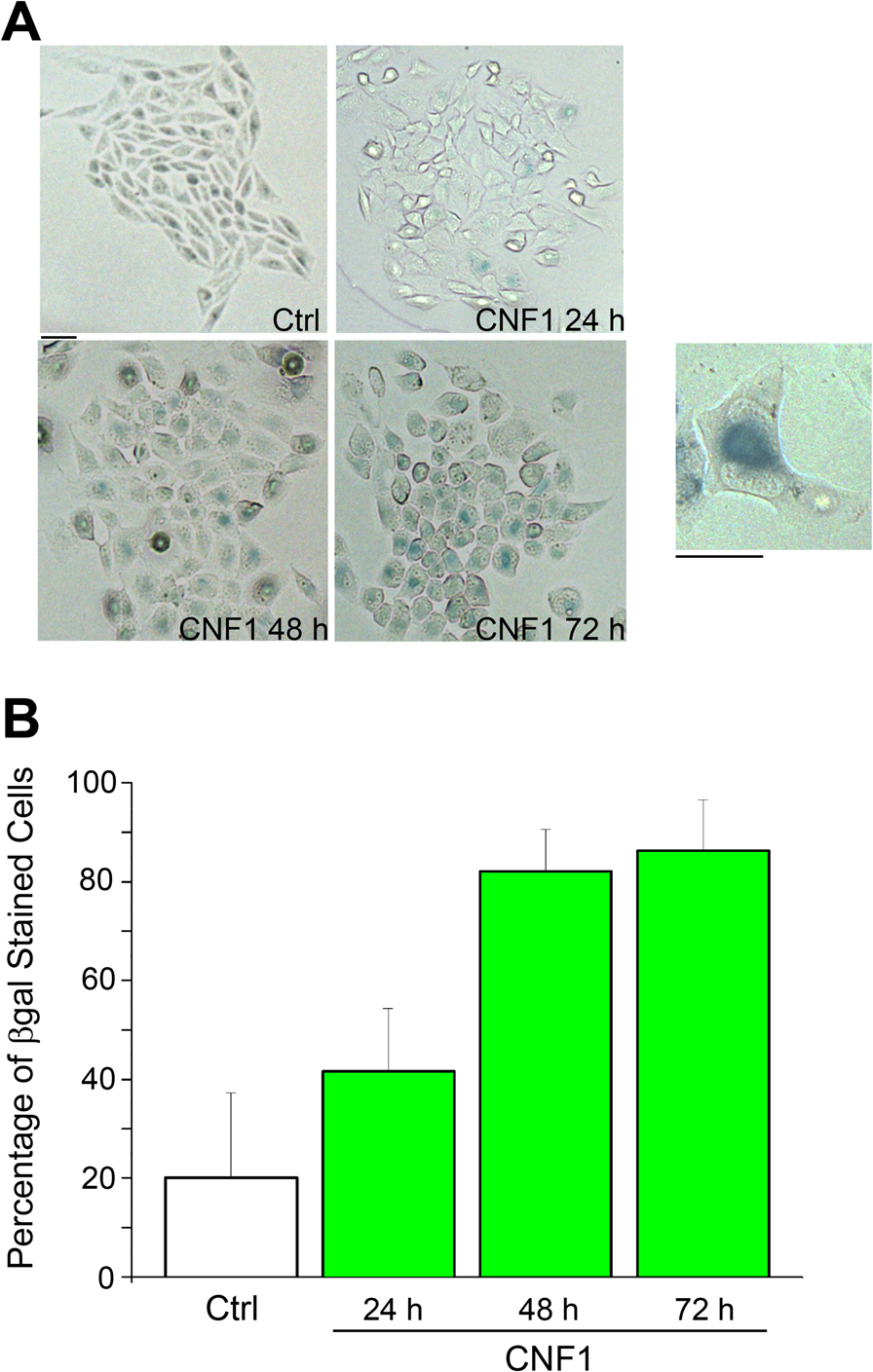
**CNF1 induces senescence in GL261 cells: SA-beta-gal staining. (A)** Representative images of SA-beta-gal staining of GL261 cells after 24 h, 48 h and 72 h of CNF1 treatment. All images were taken at 20× magnification. Scale bar = 10 μm. Inset: representative high magnification of a CNF1-treated cell at 48 hours. Scale bar = 5 μm. **(B)** Percentage of beta-gal positive cells at the different time points. Data shown are representative of three independent experiments (one way ANOVA, p < 0.0001). All data represent means ± SEM.

As indicated above, the GL261 cells treated with CNF1 are arrested in the cell proliferation and show a senescent morphology (enlargement and flattening of cells). We therefore adopted the β-Galactosidase activity, as a well accepted senescence marker, to confirm our morphological observation. The blue β-Galactosidase staining in treated cells was detectable as early as one day after treatment with the toxin (1 nM), and became intense and expressed in every cell of the culture after 72 hours. The SA-β-gal staining was not detected or barely detected in untreated control cells (Figure 5).

### CNF1-induced multinucleation in human primary tumoral cells

**Figure 6:**
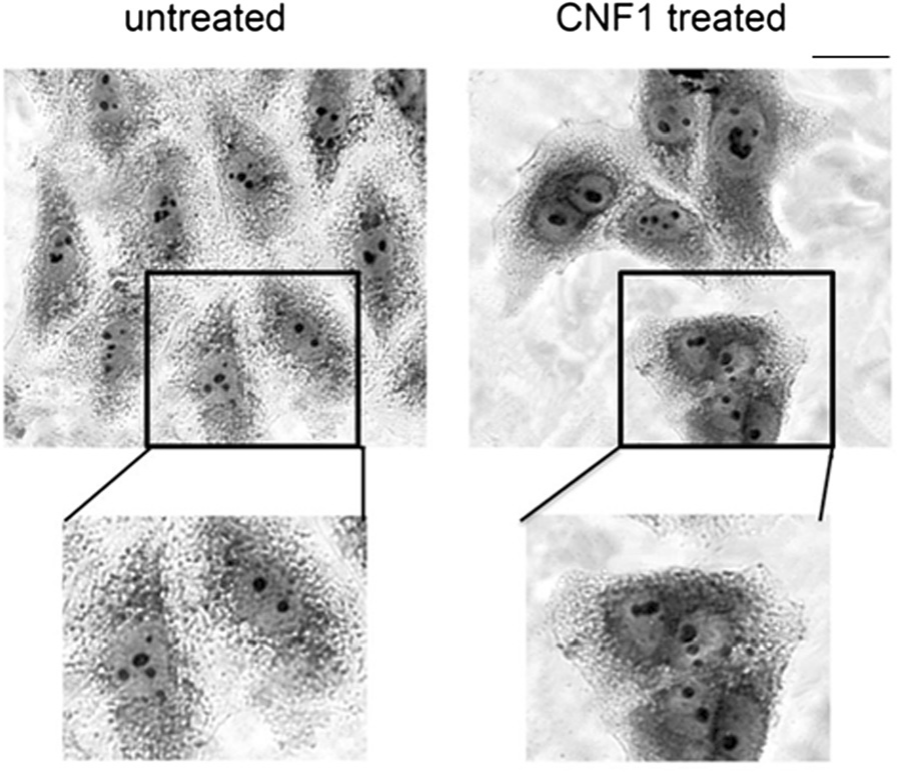
**Multinucleation in CNF1-treated human GBM cell cultures.** Crystal violet staining in untreated (left) and CNF1-treated (right) tumor-derived primary human cells. Scale bar = 4 μm. In the high magnification, note the presence of four nuclei in a CNF1-treated cell.

To obtain proof-of-principle for CNF1 action on human glioma cells, two surgical specimens were obtained from patients with GBM (WHO grade IV). CNF1 effects (18 nM) were examined in early passages (five passages) cell lines from these primary GBM specimens. Similar to GL261 cells, we observed the CNF1-induction of multinucleation in human tumor cells treated with the toxin for 9 days (Figure 6).

Overall, these data indicate a powerful cytostatic and cytotoxic effect of CNF1 on cultured glioma cells, including tumor cells from human subjects.

### Intracerebral CNF1 treatment enhances survival in a murine glioma model

Prompted by the cytostatic and cytotoxic effects of CNF1 in vitro, we tested the actions of this toxin after intracerebral inoculation of GL261 cells in adult mice. The murine GL261 model is a well accepted and widely used syngeneic transplant model for experimental glioma tumors, and reproduces many of the histopathological features of human glioma [12,13].

**Figure 7:**
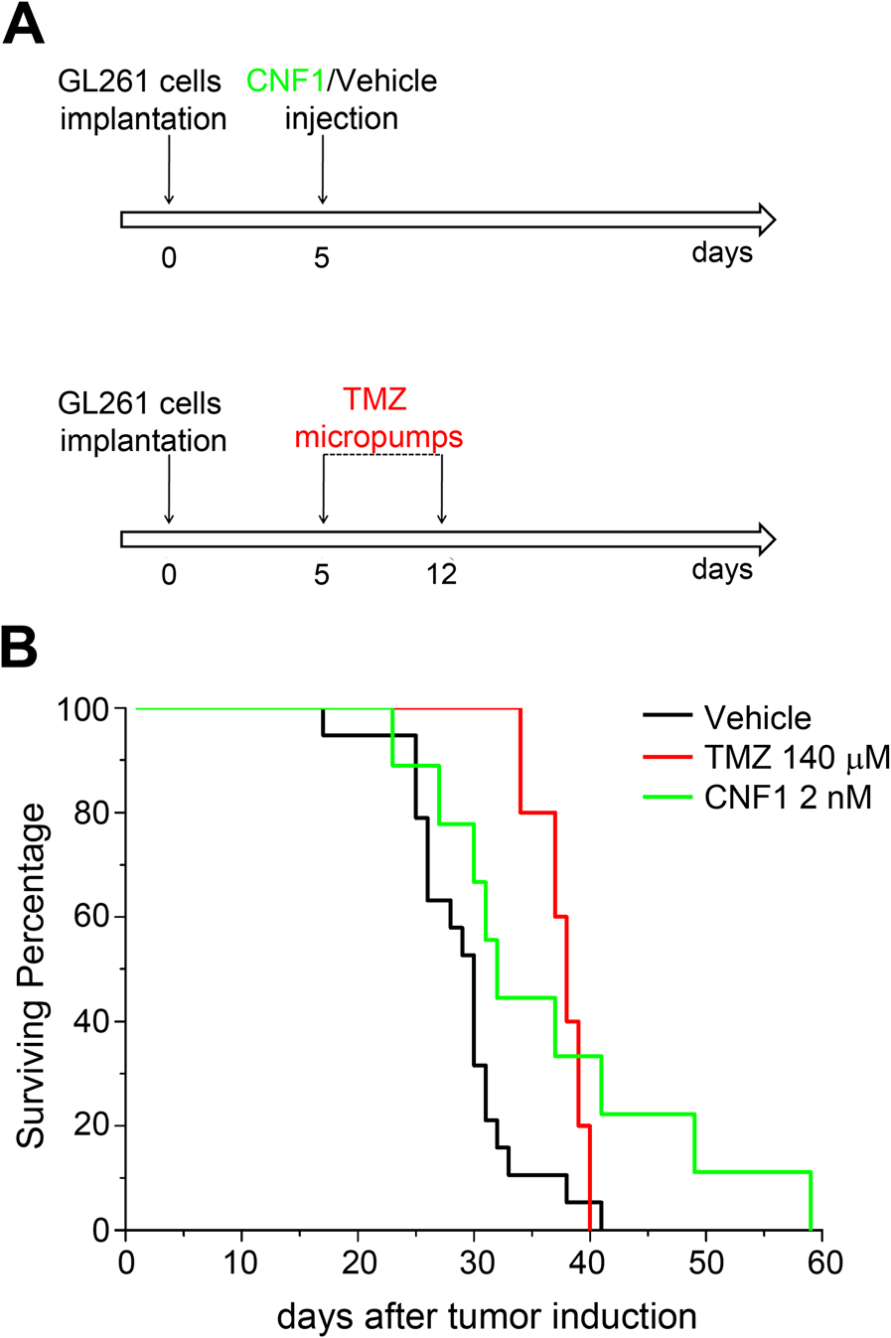
**Comparison of the survival-promoting effects of CNF1 and TMZ in the GL261 glioma model. (A)** Experimental protocol. Tumor induction by GL261 cells was either followed by a single intracerebral injection of CNF1/vehicle at day 5 (top) or by minipump delivery of TMZ from day 5 to day 12 (bottom). **(B)** Kaplan-Meier survival curves for animals implanted with GL261 cells. Compared to vehicle-infused mice (black line), CNF1 (green) and TMZ treatment (red) significantly increased survival (log Rank test followed by Holm-Sidak test, p < 0.05).

GL261 cells (40,000 cells in 2 μl) were transplanted at the level of layer VI into the occipital (visual) cortex of adult mice. Five days later, animals were injected around the transplant site with either vehicle (TRIS buffer; n = 19) or CNF1 (2 nM; n = 9; Figure 7A top). This dose of CNF1 has been previously shown to trigger a prolonged activation of Rho GTPases (particularly, Rac1) in vivo [10,11]. Kaplan-Meier survival analysis indicated a clear survival-promoting effect of CNF1 when compared to vehicle (Log Rank test, p = 0.02; Figure 7B).

### Temozolomide effect on survival of glioma-bearing mice

To directly compare the actions of CNF1 with those of classical chemotherapy, we used continuous intracerebral minipump infusions (from day 5 to day 12 post-GL261 transplant) of the alkylating agent TMZ (Figure 7A, bottom). The infusion cannula was placed directly into the cortex at a distance of 1 mm from the transplant site. Initial experiments indicated that doses of TMZ higher than 200 μM were toxic for the animals, as shown by weight loss and high mortality rate. We therefore selected a concentration of 140 μM TMZ for the minipump experiments. We found that TMZ significantly prolonged the length of survival of glioma-bearing mice (Figure 7B). In particular, the statistical analysis indicated that 2 nM CNF1 and 140 μM TMZ enhanced animal survival to a similar extent (Log Rank test, p = 0.003, followed by Holm-Sidak test; 2 nM CNF1 vs. vehicle, p = 0.02; 140 μM TMZ vs. vehicle, p = 0.002; 2 nM CNF1 vs. 140 μM TMZ, p = 0.91; Figure 7B). Lower doses of TMZ (20 μM) were completely ineffective (data not shown; n = 4).

### Higher CNF1 doses produce a dramatic increase in survival of glioma-bearing mice

**Figure 8:**
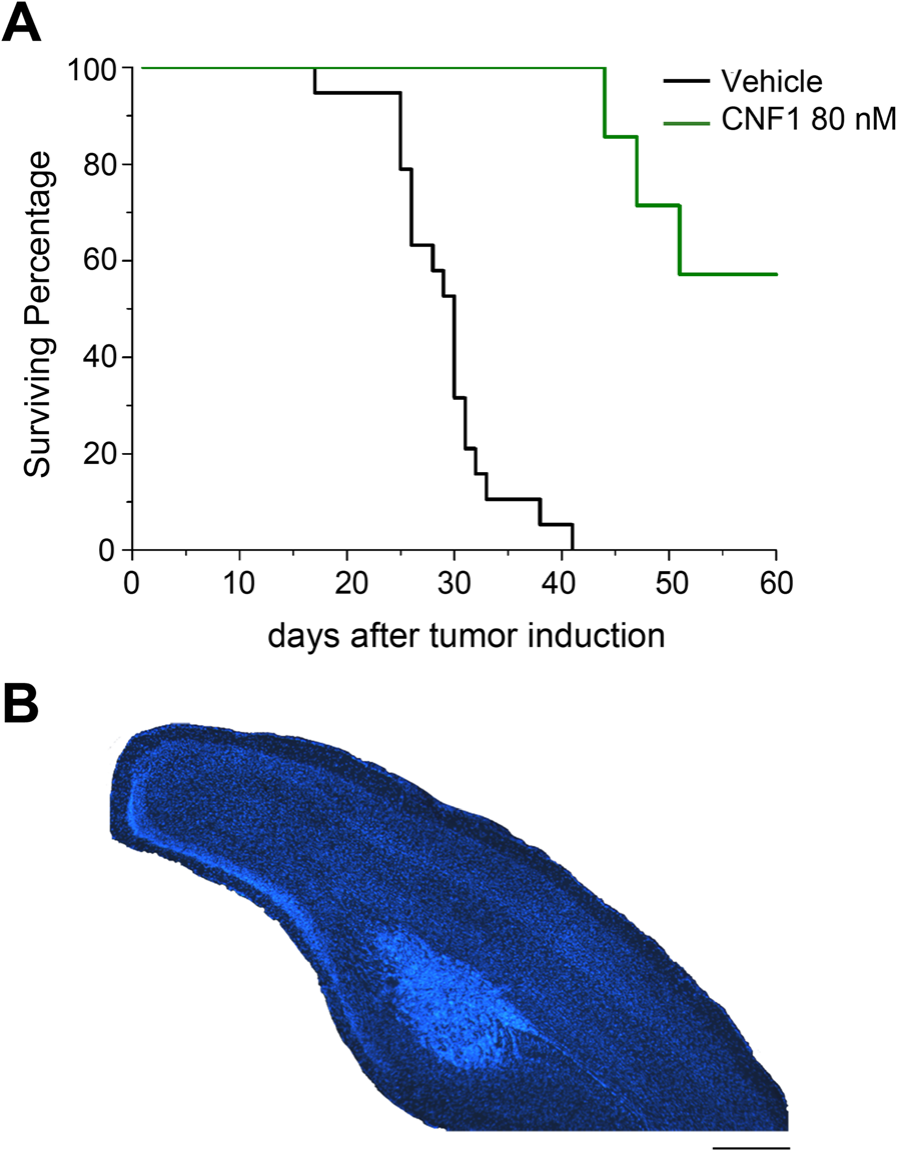
**Increasing CNF1 doses produces a dramatic enhancement of survival in glioma-bearing mice. (A)** Kaplan-Meier curves show that treating glioma-bearing mice with a higher concentration of CNF1 (80 nM) resulted in a very potent increase in mice survival. Indeed, 57% of mice injected with CNF1 80 nM were still alive 60 days after GL261 cells injection. Data for control animals are the same reported in Figure 7B. **(B)** Hoechst stained coronal section through the visual cortex of a tumor-bearing mouse treated with CNF1. The tumoral mass in the ventral part of the cortex does not affect the normal cortical layering. Scale bar = 500 μm.

We next asked whether an increase in the CNF1 dose would be effective in further enhancing animal survival. To this aim, glioma-bearing mice received a single treatment with CNF1 (80 nM; n = 7) five days after GL261 cell inoculation. We found a dramatic increase in survival, with 57% of the animals injected with 80 nM CNF1 still alive 60 days after GL261 cell inoculation (Kaplan-Meier survival analysis, CNF1 80 nM vs. vehicle, Log Rank test, p < 0.001; Figure 8A). An histopathological analysis was conducted in the CNF1-treated glioma-bearing animals surviving over 60 days. We found a small tumor located in the deep cortical layers and surrounded by apparently healthy cortical tissue (Figure 8B). These data indicate the potential of CNF1 in halting glioma growth and preserving neuronal structure.

## Discussion

The data reported in this manuscript demonstrate the therapeutic potential of a bacterial protein toxin, CNF1, in blocking proliferation of glioma cells and prolonging the survival of glioma-bearing mice. At present, there are several evidences on the use of targeted toxins to treat cancer [21]. However, the clinical efficacy of toxins has mainly been observed in hematological malignancies, but not in solid tumors, including GBM. The toxin used in this report, CNF1, is produced by certain pathogenic strains of *E. Coli* and consists of a N-terminal binding domain that interacts with membrane receptors on target cells, a middle translocation domain to enter the cytosol and a C-terminal catalytic domain [22,23]. The catalytic moiety of CNF1 activates members of the Rho GTPase family (Rho, Rac and Cdc42) by conversion (deamidation) of a single glutamine residue into glutamic acid. This aminoacid change impairs GTP hydrolysis thus locking the Rho family proteins in their GTP-bound, active state [22,23]. Depletion of activated Rho GTPases is then accomplished via ubiquitin-mediated proteasomal degradation [8].

Since Rho GTPases regulate the dynamics of the actin cytoskeleton, their activation by CNF1 triggers a rapid reorganization of F-actin [24]. In particular, proliferating cells exposed to CNF1 acquire a multinucleated phenotype, due to the inhibition of cytokinesis despite ongoing nuclear division [22,25,26]. We have observed CNF1-induced multinucleation in either GL261 glioma cells or early passage cell lines from primary GBM specimens. We have also shown that these multinucleated cells degenerate within about 15 days in vitro.

These data raise the still open question of the identification of the pathway(s) by which CNF1 causes cell degeneration. Experiments with β-Galactosidase, Annexin V and Propidium Iodide labelling allow us to conclude that senescence and then necrosis account for CNF1-induced GL261 cell death. This is consistent with the senescent-like phenotype (cell enlargement, flattening, increase in size of nuclei and nucleoli) assumed by cells treated with CNF1 (see Figure 4A, inset). At the moment, we cannot exclude the possibility that the autophagy pathway contributes to the CNF1-induced phenotype. A recent paper indicates the activation of the autophagy pathway following the treatment of glioma cells with pertussis toxin and TMZ [27]. Future studies are needed to clarify the possible role of autophagy in the antitumoral action of CNF1.

Since CNF1 is derived from *E. coli*, it might be argued that contamination by bacterial products (such as LPS) could contribute to the observed antineoplastic effects via the activation of immune responses in the brain [28]. However, this hypothesis is very unlikely, as the amount of LPS is our CNF1 preparation was found to be extremely low, in a range unable to activate macrophages (see Methods).

In GBM and in experimental glioma models, the activation of Rho GTPases (such as Rac1) has been linked to increased cell invasion [29], pointing to these molecules as key therapeutic targets. In this scenario, the dramatic effect of CNF1 on the actin cytoskeleton reorganization renders cells virtually immobile, and this might substantially enhance the anti-tumoral properties of the toxin. Indeed, we have demonstrated, in the wound migration assay, that CNF1 dramatically decreased the motility of GL261 cells, suggesting a further potential therapeutic feature of this toxin for its possible application in the treatment of glioma tumors. This aspect is important in the context of glioma treatment, because glioma cells tend to diffuse profusely into adjacent healthy tissue. It is well established that CNF1 causes assembly of F-actin in prominent stress fibers and extreme flattening of the cell body [6,24]. Since cells in motion need actin dynamics to attach and detach from the extracellular matrix, actin polymerization by CNF1 effectively renders cells stationary.

Furthermore, it is worth noting that the other key aspect of the action of CNF1 is on neuron plasticity and health [10]. This cooperates with the antitumoral effect (reduced proliferation and motility) and may lead to better preservation of neuronal function in the brain areas surrounding the tumor. Experiments to address CNF1-mediated functional sparing in glioma models are currently ongoing in our laboratory.

Importantly, a key feature of CNF1 is the rapidity of its action. We found that exposure to CNF1 for 1 hr was sufficient to halt proliferation of most of the treated cells (Figure 2). Since CNF1 is an enzyme, entry of a few toxin molecules inside a cell can lead to the modification of several Rho GTPase targets, providing a dramatic amplifying effect. Thus, even a short CNF1 exposure produced nearly maximal effects on cell proliferation. This may be important for glioma therapy, as one problem of local therapies is the rapid washout of therapeutic molecules infused in the tumor area. Moreover, the effects of CNF1 appear to persist for weeks following one single administration of the toxin [10,11,30], likely due to persistent catalytic activity of the toxin inside cells. This further strengthens the potential of CNF1 and could avoid the need for repeated drug administration.

The potential involvement of CNF1 in cell transformation is still controversial [31]. Several studies in vitro and in vivo, including the present results, demonstrate the anti-proliferative and cytotoxic effect of CNF1 in cancer cell lines [22,25,26]. Furthermore, cell transformation and tumor formation have never been observed after a single administration of CNF1 in the rodent brain (e.g. [10,11,30]).

In order to evaluate the effectivness of CNF1, we compared its action to that of a current standard chemotherapic drug, namely TMZ. Even if TMZ in experimental animal models is typically given orally, here we have chosen to administer it via an intracranial route to allow direct comparison with CNF1, which does not cross the blood–brain barrier. We found that TMZ, administered for one week via minipumps, was effective in prolonging animal survival but had a quite narrow therapeutic range, with concentrations of 20 μM being ineffective and concentrations > 200 μM being toxic for the animals. The limited efficacy of TMZ chemotherapy is in line with current clinical and experimental experience [4,32] and can be attributed to both inherent and acquired tumor drug resistance. In contrast with conventional chemotherapy, CNF1 had greater efficacy and showed no obvious side effects with increasing doses. Remarkably, more than half of the animals treated with 80 nM CNF1 survived for at least 60 days following glioma cell inoculation. One important aspect of CNF1 is its long-lasting action, as one single intracerebral administration leads to Rho GTPase activation for at least 10–28 days [10,11,30].

## Conclusions

In summary, we have exploited the remarkable properties of CNF1 for interfering with glioma proliferation in vitro and in vivo. Specifically, we demonstrated in vitro that: (i) CNF1 blocks proliferation of GL261 cells, (ii) induces cell senescence and death, (ii) inhibits migration of tumor cells in the wound assay. In vivo, CNF1 was more effective than TMZ in prolonging survival of tumor-bearing mice. Given these antitumoral actions, and the ability of CNF1 to enhance learning, memory and plasticity in the intact and diseased brain [10,11,30], the present data suggest that CNF1 might hinder glioma growth and at the same time preserve neuronal responses in the peritumoral area.

## Abbreviations

CNF1: Cytotoxic necrotizing factor 1; TMZ: Temozolomide; GBM: Glioblastoma multiforme; WHO: World Health Organization; ANOVA: Analysis of variance; SEM: Standard error of the mean; SA-β-gal: Senescence-associated β-galactosidase; LPS: Lipopolysaccharide.

## Competing interest

The authors declare that they have no competing interest.

## Authors’ contributions

EV carried out all *in vivo* experiments, performed the statistical analysis and drafted the manuscript. AP carried out all *in vitro* experiments, performed the statistical analysis and drafted the manuscript. CC partecipated in the *in vivo* experiments. SL performed the *in vitro* experiments with human glioblastoma cells. EC performed the cell death assays. AF and CF provided CNF1 toxin. NB and RV provided human biopsies of glioblastoma multiforme. MCa and MCo conceived of the study, and participated in its design and coordination and drafted the manuscript. All authors read and approved the final manuscript.

## Pre-publication history

The pre-publication history for this paper can be accessed here:

http://www.biomedcentral.com/1471-2407/14/449/prepub
